# Oleic Acid Metabolism *via* a Conserved Cytochrome P450 System-Mediated ω-Hydroxylation in the Bark Beetle-Associated Fungus *Grosmannia clavigera*

**DOI:** 10.1371/journal.pone.0120119

**Published:** 2015-03-20

**Authors:** Metka Novak, Ljerka Lah, Martin Šala, Jure Stojan, Joerg Bohlmann, Radovan Komel

**Affiliations:** 1 National Institute of Chemistry, Hajdrihova 19, SI-1000, Ljubljana, Slovenia; 2 Institute of Biochemistry, Faculty of Medicine, University of Ljubljana, Vrazov trg 2, SI-1000, Ljubljana, Slovenia; 3 Michael Smith Laboratories, University of British Columbia, Vancouver, BC V6T 1Z4, Canada; Cincinnati Childrens Hospital Medical Center, UNITED STATES

## Abstract

The bark beetle-associated fungus *Grosmannia clavigera* participates in the large-scale destruction of pine forests. In the tree, it must tolerate saturating levels of toxic conifer defense chemicals (e.g. monoterpenes). The fungus can metabolize some of these compounds through the ß-oxidation pathway and use them as a source of carbon. It also uses carbon from pine triglycerides, where oleic acid is the most common fatty acid. High levels of free fatty acids, however, are toxic and can cause additional stress during host colonization. Fatty acids induce expression of neighboring genes encoding a cytochrome P450 (CYP630B18) and its redox partner, cytochrome P450 reductase (CPR2). The aim of this work was to study the function of this novel P450 system. Using LC/MS, we biochemically characterized CYP630 as a highly specific oleic acid ω-hydroxylase. We explain oleic acid specificity using protein interaction modeling. Our results underscore the importance of ω-oxidation when the main ß-oxidation pathway may be overwhelmed by other substrates such as host terpenoid compounds. Because this CYP-CPR gene cluster is evolutionarily conserved, our work has implications for metabolism studies in other fungi.

## Introduction

The ascomycete fungus *Grosmannia clavigera* (*Gs*) is associated with the mountain pine beetle (MPB; *Dendroctonus ponderosae*) and actively participates in the large-scale destruction of lodgepole pine forests in western North America [1]. To successfully colonize the tree, the fungus must optimize sequestration of carbon mainly from phloem and sapwood carbohydrates, fats and proteins, and retrieve nitrogen and other essential micro-nutrients [2]. In addition, it must overcome host conifer chemical defenses. Among these, one of the most abundant groups of chemicals are terpenoids [3]. Several studies have focused on molecular mechanisms of monoterpene resistance in *Gs*, which include monoterpene export through ABC transporter activity, and enzymatic monoterpene detoxification and channeling into metabolic pathways [4, 5]. Transcriptome analysis of *Gs* showed that genes up-regulated in response to monoterpenes included acetyl-CoA-acyltransferase, alcohol dehydrogenase, and genes involved in fatty acid metabolism and ß-oxidation pathways; many of which are localized in the same genomic region [4]. Molecular studies, and observed survival and growth of *Gs* in the presence of saturating levels of monoterpenes show that this fungus can utilize host monoterpenes as a carbon source.

Another important source of carbon for the fungus are conifer triglycerides, where the most common esterified fatty acids in fatty acyl-glycerols are oleic acid and linoleic acid [6]. Triglycerides and fatty acids comprise around 2.5% dry weight of lodgepole pine sapwood [7]. Oleic acid is also one of the most abundant free fatty acids in pine stems [6, 8, 9]. Like monoterpenes, excessive amounts of free fatty acids are toxic to the fungal cell and have been shown to affect membrane fluidity and inhibit membrane protein activity [10–13]. Transcriptome profiling of *Gs* grown on these compounds showed induction of genes involved in fatty acid ß-oxidation [14, 15], as well as extracellular lipases possibly involved in triglyceride hydrolysis [4]. Conspicuously, two of the most highly induced genes were co-localized in the genome and encode a cytochrome P450 (CYP) and its redox partner, cytochrome P450 reductase (CPR): *Gs*CYP630 and *Gs*CPR2 [4, 14, 15]. Further, the expression of two genes adjacent to this CYP cluster that code for a transcription factor and a transporter protein was also induced. The CYP630-CPR gene cluster has also been identified in genomes of other fungal species [16–20]. However, to the best of our knowledge, its biochemical and biological functions have not yet been characterized.

CYPs are ubiquitously present heme-thiolate proteins, which oxidize a vast array of endogenous and exogenous substrates. Fungi possess numerous CYPs, most of which have not been functionally characterized. Knowledge of functions of CYPs in plant-pathogenic fungi is important as these enzymes may contribute to detoxification of plant defenses or to biosynthesis of mycotoxins that affect the host or other organisms [21]. CYPs localized in the endoplasmic reticulum are known to be involved in ω-hydroxylation of fatty acids, which is normally a minor pathway for fatty acid degradation. However, when ß-oxidation is ineffective, ω-hydroxylation becomes more important [22]. In fungi, functionally characterized CYPs involved in fatty acid ω-hydroxylation mainly belong to the CYP52 and CYP505 families [23–28]. Both CYP families were present in the common ancestor of fungi, and are frequent in the ascomycetes and basidiomycetes. CYP52 is one of the most frequent families, particularly in both ascomycetous filamentous fungi and yeasts [29]. No CYP52 family member has been identified in the *Gs* genome, and the expression of CYP505 on tested fatty acid substrates is low [14].

In the present study we functionally characterized *Gs*CYP630B18 in combination with two possible CPR redox partners, *Gs*CPR1 and *Gs*CPR2, as a highly specific oleic acid ω-hydroxylase. We tested the catalytic properties of these enzymes in reconstituted systems using saturated and non-saturated fatty acids of different lengths as substrates and identified reaction products. We used protein-protein interaction modeling to additionally explain our experimental results. Furthermore, to provide a broader overview of the taxonomic distribution of the CYP630-CPR2 cluster, specific to fungi, we examined its presence in the genomes of filamentous ascomycetes. Finally, we proposed a pathway model for the role of CYP630B18 in fatty acid ω-oxidation and hypothesized on the importance of this pathway in pine tree colonization by *Gs*.

## Materials and Methods

### cDNA cloning of GsCYP630B18, GsCPR1 and GsCPR2

All bacterial and yeast strains, plasmids, primers and PCR conditions used in this study are listed in S1 Table. Coding sequences for *Gs*CYP630B18, *Gs*CPR1 and *Gs*CPR2 from *Gs* cDNA were amplified using the Phusion Hot Start II High-Fidelity DNA Polymerase (Thermo Scientific). Purified *Gs*CYP630B18 PCR products were inserrted into the pET17b vector (Novagen, USA), and *Gs*CPR1, *Gs*CPR2 products into the pYeDP60U expression plasmid [30].

### Expression and purification of GsCYP63018B and GsCPRs

The proteins were expressed and purified as described previously [16]. Membrane fractions were resuspended in 100 mM Tris acetate (pH 7.4)/0.5 M sucrose/0.1 mM EDTA, and stored at -80°C. For expression of CPRs in yeast, single transformants were grown on SD-URA:URA dropout medium with D-glucose as a carbon source with amino acids (S2 Table), and sub-cultured overnight in 5 ml of SD-URA dropout medium. 50 ml cultures into 200 ml YPD-E growth medium (YP-Yeast extract, Bacto peptone, 10% (20%) D-glucose, 5% ethanol; BD, USA) were transferred and incubated for 24 h at 30°C. Expression was induced for 18 h at 30°C in YPGal medium (YP-Yeast extract, Bacto peptone, 10% (20%) galactose; BD, USA). Yeast microsomal membrane fractions were prepared from 200 ml cultures at 4°C according to [31].

### GsCYP630B18 reconstitution systems

The activity of *Gs*CYP630B18 was tested in reconstitution systems with *Gs*CPR1 and *Gs*CPR2 (RS1 and RS2, respectively) and substrates as described previously [32]. For RS1 and RS2, a 0.2 ml membrane fraction of approximately 16.8 μM *Gs*CYP630B18 and 0.2 ml of the yeast microsomes of 13 μM *Gs*CPR1 and *Gs*CPR2 for RS1 and RS2, respectively, was prepared in 50 mM Tris/HCl (pH 7.4)/0.1 mM EDTA/10% glycerol. The reactions were initiated by adding 0.1 M NADPH and substrate (50 μM oleic acid (C18:1), linoleic acid (C18:2), stearic acid (C18:0), palmitic acid (C16:0), palmitoleic acid (C16:1), myristic acid (C14:0), lauric acid (C12:0), capric acid (C10:0) or arachidonic acid (C20:4)). After one hour of incubation at RT the reactions were stopped. We extracted the organic product with methanol and analyzed it with LC/MS.

### Metabolite identification

The activity of *Gs*CYP630B18 was measured by monitoring product formation on the LC Agilent 1100 Series system (Agilent Technologies, USA) using a YMC-ODS-AQ chromatographic column coupled to an Applied Biosystems 4000 hybrid linear ion trap-triple-quadrupole mass spectrometer (QTrap; AB SCIEX, Concord, ON, Canada). For the analysis of fatty acids, 10 μL of the enzyme reaction extract was injected, separated and analyzed as described [33] using a gradient program with a two solvent system (S3 Table) and negative mode ESI ion source for ionization. For statistical analysis, a Welch’s *t*-test with unequal variances using XCMS software was calculated [34, 35]. A P-value < 0.05 indicated significant differences between controls and samples. The product was confirmed using high-resolution mass spectrometry with Agilent technologies 1260 infinity LC system and Agilent technologies 6224 TOF/LC/MS instrument. The product was fragmented on the Agilent 1100 series LC/MSD ion trap XCT Plus system. The fragmentation pattern was analyzed with the ACD/MS Fragmenter software (ACD/MS Fragmenter v.12, Advanced Chemistry Development, Inc. (www.acdlabs.com; Toronto, ON, Canada). All fragmentation data were generated using a collision energy of 20 volts.

### Activity and kinetics of GsCPRs

The activity of membrane-bound CPRs was assayed using the protocol optimized previously [16]. To test the catalytic power of the two CPRs, the reduction of ferricyanide was followed in the presence of saturating 100 mM NADPH on a UV-2450 Shimadzu spectrophotometer at 420 nm [16]. The initial kinetic parameters were evaluated using ENZO [36]. Based on constructed curves, equation 1 was set to:

$$\begin{array}{c} E+NADPH\\ k_{0}↓↑k_{1}\\ E-NADPH+Ferricyanide\\ k_{2}↓↑k_{3}\\ E-NADP^{+}+Ferricyanide\\ k_{4}↑↓k_{5}\\ K+NADP^{+} \end{array}$$

and the modified classical Michaelis—Menten equation 2 was set to:

$$v=\frac{k_{cat}\cdot A_{0}\cdot E_{0}}{A_{0}+K_{M}}$$

where: *v*—reaction rate, *E*_*0*_—enzyme concentration, and *A*_*0*_—substrate concentration. *K*_M_ ($\frac{k_{1}\cdot k_{3}+k_{1}\cdot k_{4}+k_{2}\cdot k_{4}}{k_{0}\cdot \left(k_{2}+k_{3}+k_{4}\right)}$)and *k*_cat_ ($\frac{k_{2}\cdot k_{4}}{k_{2}+k_{3}+k_{4}}$) represent the Michaelis and the catalytic constants, respectively. The reduction of ferricyanide to ferrocyanide was quantitated by the loss of absorbance at 420 nm. In our plots, we used point zero as the baseline and normalized all points relative to this baseline [37, 38]. The calculated AU units were therefore negative.

### Modeling and docking of GsCPR2 and GsCYP630B18

The FMN-binding domain of GsCPR2 was modeled using the structure of human-yeast chimeric CPR (PDB ID: 3FJO) as template, as described previously [32]. Briefly, the two sequences were aligned (residues 48–214 from 3FJO with residues 27–199 from GsCPR2), the residues mutated accordingly with the Whatif molecular modeling tool (35.3% identity) and the gaps manipulated manually (two insertions with two residues each) (Vriend, 1990). After adding FMN in its original orientation to 3FJO and structure optimization, 13559 explicit water molecules were added and a 1 ns constant pressure and temperature (CPT) dynamic simulation (300K, 1bar, time step 1fs) invoking the EWALD summation for calculating the electrostatic interactions was run using CHARMM molecular simulation software [39]. CYP638B18 was modeled following the same protocol and using crystal structures of two human drug-metabolizing cytochromes as templates (PDB IDs: 1Z10 and 1PQ2).

The last frame of each dynamic run was optimized (50 steps of steepest descent, followed by 50 ABNR steps) and used as input for protein-protein docking. The initial orientation of the two structures was chosen on the basis of the binding site searching algorithm, implemented in ProBis (http://probis.cmm.ki.si/) [40]. Subsequently, the complex was submitted to the RosettaDock server for local protein-protein docking (http://rosettadock.graylab.jhu.edu/viewjob?id=4294) [41]. From the several suggested structures, the one that predicted the shortest FMN—heme distance was selected. Further modeling comprised another 1 ns CPT-EWALD dynamic simulation of the complex. Finally, a phospholipid bilayer was added, and the complex, oriented with the N-terminal regions and the putative CYP638B18 active site entrance towards the membrane.

In parallel, oleic and stearic acids were docked into the active site by positioning their ω-methyl group at 3.5 A distance from the iron and the aliphatic chain towards the membrane-oriented surface. After structure optimization, a short 0.2 ns CPT-EWALD dynamic simulation was run. For comparison, the movements were analyzed with CHARMM. In all simulations, the latest CHARMM force fields were used and the complexes visualized using VMD, a program for displaying, animating, and analyzing biomolecular systems [42].

### GsCYP630-CPR2 cluster conservation in Pezizomycotina

The Pezizomycotina genomes deposited in the NCBI and JGI databases (http://www.ncbi.nlm.nih.gov/ and http://www.jgi.doe.gov/, respectively) were mined with the protein BLAST program [43] using protein sequences of *Gs*CYP630B18 (Genbank ID: EFX05103) and *Gs*CPR2 (Genbank ID: EFX05108) as separate queries. Other fungal taxa were checked for the presence of the cluster, however they were not investigated thoroughly, since the CPR1-CPR2 gene duplication event was independent in Pezizomycotina [19]. Genomes, where hits to both queries with low E-values (E-value cutoff = 1*e*-50) were located adjacently on the same contig were considered to have retained the CPR2-CYP630 gene cluster. Genomes with significant hits to either of the queries, but not located on the same contig were excluded from the analysis.

## Results

### GsCYP630B18 is an oleic acid ω-hydroxylase

We expressed the cDNA of *Gs*CYP630B18 in *E*. *coli* [16]. Optimal expression was achieved in culture induced with IPTG at OD600 0.6–0.8 and then grown for 24 h at 30°C. The average expression level of *Gs*CYP630B18, detected in the purified membrane fraction was 3.1 μmol l^-1^. Protein homogeneity was verified *via* SDS-PAGE, showing a 61 kDa band corresponding to *Gs*CYP630B1. We successfully expressed *Gs*CPR1 and *Gs*CPR2 genes in yeast, under the galactose-inducible GAL10-CYC1 hybrid promoter. Average protein expression levels were 10.3 μmol l^-1^ for CPR1 (76.2 kDa) and 15.4 μmol l^-1^ for CPR2 (77.3 kDa), which is within the range of protein expression values reported for other fungal CPRs [16, 44, 45].

For functional characterization, we expressed *Gs*CYP630B18 in two different reconstitution systems (RS), RS1 and RS2, with *Gs*CPR1 and *Gs*CPR2, respectively. We tested substrate conversion and specificity of RS1 or RS2 with saturated and unsaturated fatty acids of different chain lengths: oleic acid (C18:1), linoleic acid (C18:2), stearic acid (C18:0), palmitic acid (C16:0), palmitoleic acid (C16:1), myristic acid (C14:0), lauric acid (C12:0), capric acid (C10:0) and arachidonic acid (C20:4). Product formation was analyzed in methanol extracts by LC/MS. Both CPRs supported activity of *Gs*CYP630B18 in *in vitro* assays, despite having different catalytic efficiencies. CYP630B18 converted oleic acid, but none of the other tested compounds (S1 and S2 Figs.).

**Fig 1:**
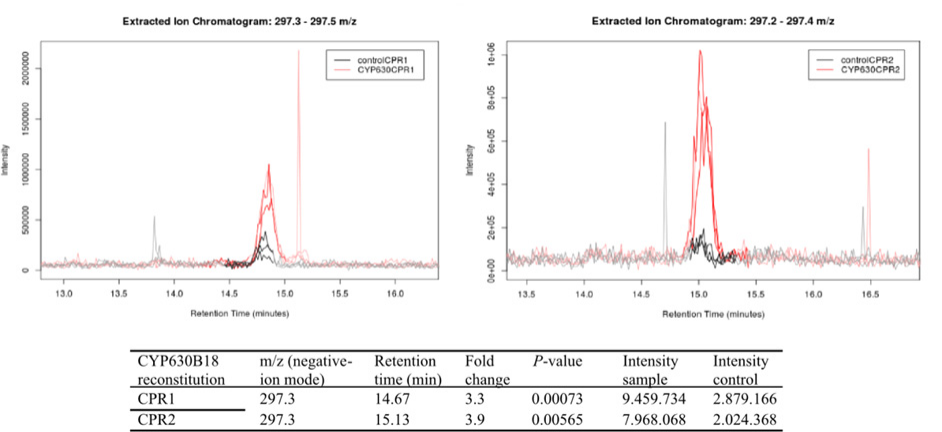
Oleic acid oxidation by *Gs*CYP630B18 in combination with *Gs*CPR1 or *Gs*CPR2. Extracted ion chromatograms (297.2–297.4 m/z, run in negative-ion ESI-MS) for oleic acid conversion by GsCYP630B18 in combination with GsCPR1 or GsCPR2 with the highest peak (m/z 297.3) identified as 18-hydroxyoleic acid. Darkened lines indicate where the peaks were integrated for relative ion intensity comparison. Samples GsCYP630 with GsCPR1 (top left panel) and GsCYP630 with GsCPR2 (top right panel) are shown in red. Controls (empty E. coli membrane fractions with GsCPR1or GsCPR2) are shown in black. Significant differences for the major product peak between controls and samples were indicated by P-value (P < 0.05, n = 3). Additional data for each analysis are shown below the LC/MS traces as m/z value, retention time, fold change as ratio of mean intensities between samples and controls, P-value, and peak intensity as average feature intensity within sample/control class.

**Fig 2:**
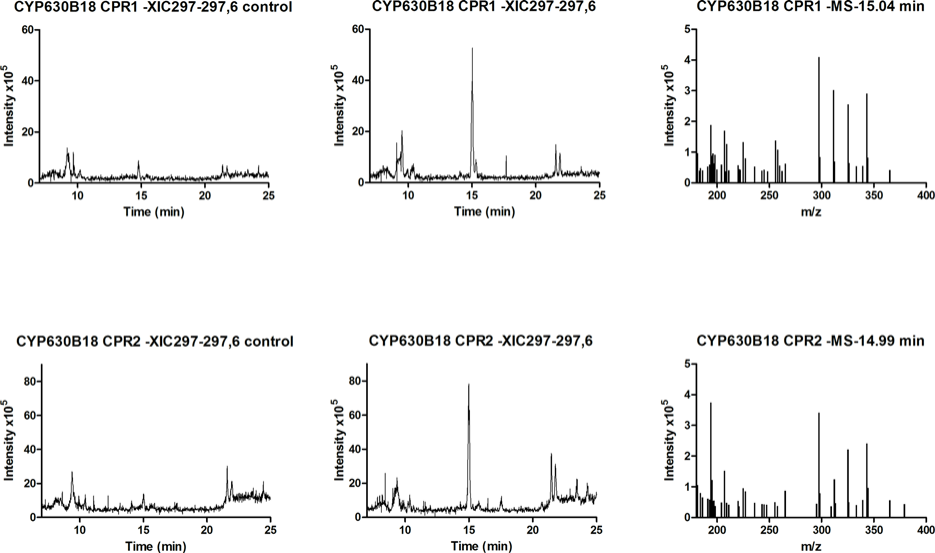
Single extracted ion chromatograms of RS1 and RS2 products. Extracted ion chromatograms (297.2–297.6 *m/z* run in negative-ion ESI) from LC/MS analyses for *Gs*CYP630B18 activity in RS1 and RS2 with oleic acid as substrate. Empty *E*. *coli* membrane fractions were used as control. Mass spectra representing hydroxyoleic acid peaks with RT 15.04 and 14.99 min are shown on the right.

The major peak of a single product of molecular weight 298 in the extracted ion chromatogram (Fig. 1) was identified by its fragmentation pattern as 18-hydroxyoleic acid which could be formed by either epoxidation or hydroxylation of oleic acid (S3 Fig., S4 Table). TOF/LC/MS confirmed the enzyme product as C_18_H_33_O_3_ (calculated for hydroxyoleic acid C_18_H_33_O_3_^-^ [M-H]^-^: 297.2508; found: 297.2435; difference 0.1ppm). Control microsomes without *Gs*CYP630B18 did not oxidize any of the fatty acids (Fig. 2). Welch’s t-test showed highly significant differences between controls and samples for the presence of the major product peak. P-values for RS1 and RS2 were 0.0007 and 0.0057, respectively (Fig. 1).

### GsCPR1 and GsCPR2 have different catalytic properties

**Fig 3:**
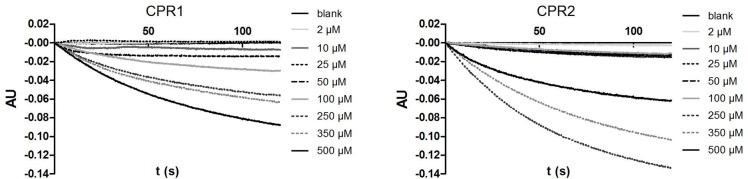
Standard plots for determining ferricyanide reduction kinetics catalyzed by *Gs*CPR1 or *Gs*CPR2 in the presence of NADPH. The CPR-catalyzed reduction of ferricyanide in concentrations between 2 μM and 500 μM at 420 nm in the presence of saturating 100 mM NADPH in 100 mM potassium phosphate (pH 7.6). Different concentrations of ferricyanide are represented in shades of grey and dashed lines. Decreasing absorbance at 420 nm, quantifying the reduction of ferricyanide to ferrocyanide, was plotted by normalizing all points relative to the point zero baseline.

We used a standard cytochrome c reduction assay to test the NADPH-dependent activity of the yeast microsomal fractions containing GsCPR1 or GsCPR2. While both CPRs reduced cytochrome c, GsCPR2 was more active than GsCPR1. Membrane fractions of yeast cells with the empty plasmid used as control showed substantially lower cytochrome c reducing activity (Table 1). CPR1 and CPR2 had comparable affinities for the substrate as revealed by their K_M_ values, which were 274 μM and 256 μM, respectively (Fig. 3, Table 2). The turnover number k_cat_ was 37 s^-1^ for GsCPR1 and 989 s^-1^ for GsCPR2. The enzyme catalytic efficiency, or the specificity constant (k_cat_/K_M_) was thus about thirty times higher for GsCPR2 (3.87 x 10^6^ M^-1^ s^-1^) than for GsCPR1 (0,14 x 10^6^ M^-1^ s^-1^).

**Table 1:** Enzyme activity of *Gs*CPR1 and *Gs*CPR2.

Enzyme	Activity ± SD (EU/ml)
*Gs*CPR1^m^	1.540 ± 0.140
*Gs*CPR2^m^	2.380 ± 0.320
Positive control	0.200 ± 0.020
Negative control	0.010 ± 0.003

EU—one enzyme unit of CPR reduces 1.0 μmol oxidized cytochrome c per minute in the presence of 100 mM NADPH at pH 7.8 and 25°C.

m—membrane-bound

Positive control—rabbit liver CPR

Negative control—membrane fraction of yeast strain expressing empty plasmid pYEDP60U

**Table 2:** Calculated kinetic parameters for *Gs*CPR1 and *Gs*CPR2.

Kinetic parameters	Values (s^-1^)	± SD
*Gs*CPR1		
k_0_	6.14 · 10^6^	0.37 · 10^6^
k_1_	0.86	0.06
k_2_	0.61 · 10^6^	909.65
k_3_	16.68 · 10^6^	0.33 · 10^6^
k_4_	37.14	0.76
k_5_	2.86 · 10^6^	1.76 · 10^6^
*Gs*CPR2		
k_0_	5.07 · 10^6^	0.36 · 10^6^
k_1_	13.27	0.72
k_2_	0.26 · 10^6^	93.23
k_3_	6.65 · 10^6^	0.00
k_4_	1028.60	48.80
k_5_	9.74 · 10^6^	0.00

### The CYP630-CPR2 gene cluster is specific to Pezizomycotina

**Fig 4:**
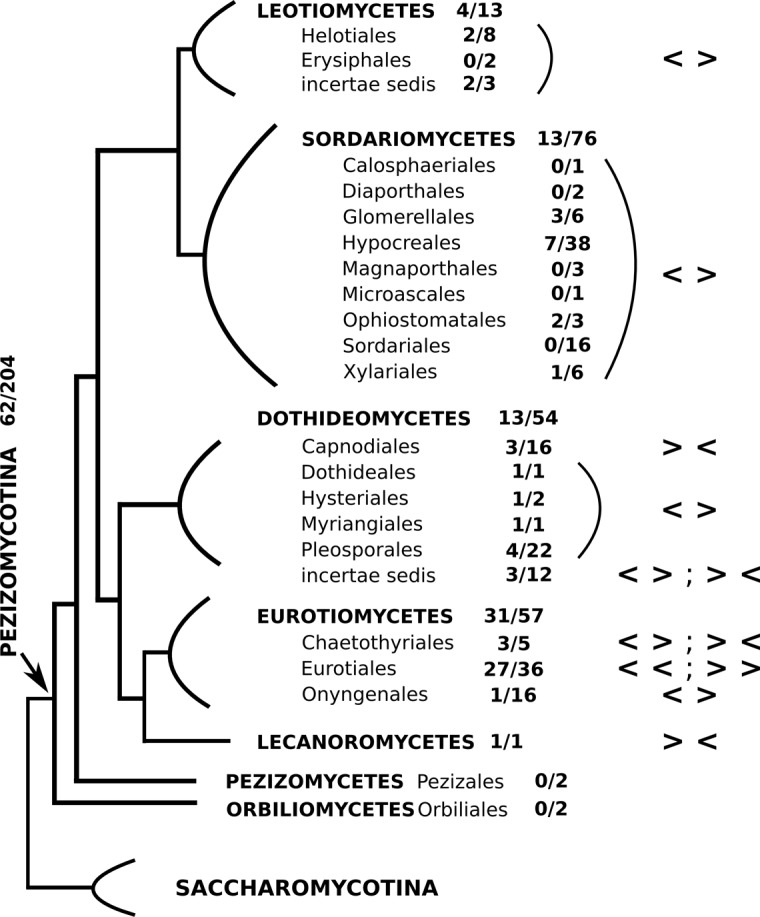
Schematic representation of selective conservation of the CYP630-CPR2 gene cluster in Pezizomycotina. The presence of the gene cluster found in seven Pezizomycotina classes is given as a fraction of the species that the gene cluster was identified in relative to all the species of a given class whose genomes were searched. Homologs of either gene were not identified outside of Pezizomycotina. The relative orientation of both genes is given (< >—divergent; > <—convergent, << or >>—co-oriented) [47]. The phylogeny was modeled after [48].

Database mining revealed that the adjacent gene organization of orthologous CYP630 and CPR2 genes is conserved in around 30% of the 204 investigated genomes of Pezizomycotina fungi that belong to several Pezizomycotina classes (Fig. 4). The CYP630-CPR2 cluster was identified in over half of the Eurotiomycetes, mostly due to its presence in three quarters of the Eurotiales genomes where both genes were oriented in the same direction (co-oriented)[46]. The cluster was further found in nearly a quarter of Dothideomycete genomes and 17% of Sordariomycete genomes. In Dothideomycetes, the two genes were oriented in the opposite directions on the DNA strand with adjacent promotor regions (divergently), with the exception of the taxa Capnodiales and *incertae sedis*. In Sordariomycetes, the gene orientation relative to one another was exclusively divergent. No homologs of either gene were found in basal clades of Pezizomycotina (Orbilomycetes and Pezizomycetes), or ascomycetous yeasts (Saccharomycotina).

## Discussion

### Putative biological role of GsCYP630B18 as a specific oleic acid hydroxylase

To the best of our knowledge, functions of CYP630 family enzymes have not previously been reported, and the biological roles of GsCYP630B18 and GsCPR2 homologs are not known in fungi. In this study, we have biochemically characterized the first fungal CYP630 family member, peculiar to the Pezizomycotina taxon, as an ω-hydroxylase, highly specific for oleic acid conversion. In fungi, the well-characterized CYP52 family (P450Alk) contains members that can metabolize n-alkanes and fatty acids [35]. CYP52 enzymes are present in filamentous ascomycetes, several of which are plant pathogens, and putatively involved in the penetration of host cuticle, which is made up of hydrocarbons [28, 49]. Up to 12 CYP52 paralogs have been identified in plant symbiontic fungi from the genus *Trichoderma* [36, 50]. The isozymes of this multigene CYP family abundant in n-alkane-assimilating ascomycetous yeasts have different but often overlapping substrate specificities ([28], and references therein). They are capable of converting exogenous n-alkanes, alkenes, fatty alcohols, and fatty acids of various chain lengths. Enzymes belonging to completely different CYP families, CYP63A2 and CYP5136A3, were functionally characterized in the basidiomycete *Phanerochaete chrysosporium* [51, 52]. These catalytically diverse CYPs are capable of converting polycyclic aromatic hydrocarbons, alkylphenols and alkanes. Structural modeling revealed an unusually large active site cavity in CYP63A2, which was also found to act as an alkane ω-hydroxylase [51]. This is in contrast to the comparatively narrow substrate specificity of CYP630B18 and its active site cavity modeled in this study, which can specifically accommodate oleic acid.

Enzymes of the CYP505 family, where the CYP and CPR are fused into one protein encoded by a single ORF, have also been characterized as fatty acid ω-hydroxylases, however their physiological role is not clear [53–55]. In *Fusarium verticillioides*, CYP505 is part of a secondary metabolite gene cluster and implicated in mycotoxin biosynthesis [56]. Of the above listed CYP families, only a CYP505 ortholog is present in *Gs*. It is not localized in a biosynthetic gene cluster, nor is its expression highly induced on fatty acid substrates, so it is difficult to hypothesize on its function [14].

**Fig 5:**
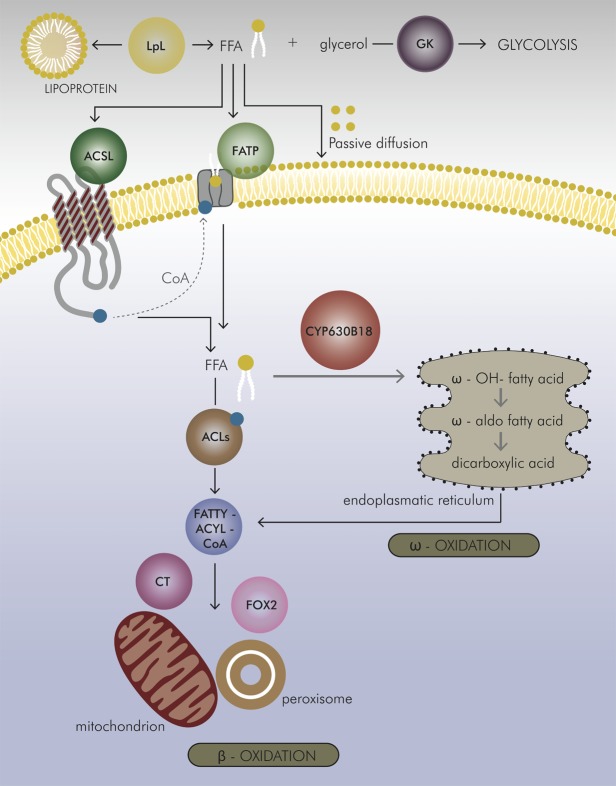
Proposed model for the role of *Gs*CYP630B18 in fatty acid oxidation. The putative involvement of *Gs*CYP630B18 is indicated by grey arrows. Triglycerides from the host plant in the lipoprotein are broken down to free fatty acids (FFA) and glycerol by the action of the lipoprotein lipase (LpL) [57]. Glycerol is further phosphorylated by glycerol kinase (GK) [58, 59] and enters glycolysis. On the cytosolic site, FFAs are activated and coupled to coenzyme A (CoA) by the catalysis of long-chain fatty acyl-CoA synthetases (ACSLs) [60] or by different fatty acid transporter proteins (FATPs) [61]. The transfer through the plasma membrane occurs by a protein-mediated mechanism. In the cell, FFAs can act at different sub-cellular localizations and have functions in energy generation and storage, membrane synthesis, protein modification, and activation of nuclear transcription factors [62]. Oxidation of FFA in fungi occurs mainly through β-oxidation in the mitochondrial matrix or peroxisomes, or through the ω-oxidation pathway in the endoplasmic reticulum. Several acyl-CoA ligases (ACLs) [63], involved in fatty acid metabolism, then attach CoA to the ends of fatty acids to form fatty-acyl-CoA. Fatty-acyl-CoA can pass through the outer mitochondrial membrane, but requires carnitine acetyl transferase (CT) [64] to cross the inner membrane. The multifunctional β-oxidation enzyme FOX2[65, 66] was induced in *Gs* mycelia grown on fatty acids, indicating possible peroxisomal oxidation. In ω-oxidation, the hydroxylase reaction is catalyzed by CYP630 (red bubble) and its redox partner CPR 2. Dicarboxylic acids are then subject to further β-oxidation. Gene IDs and respective changes in transcript abundance in *Gs* grown on fatty acids are given in Table 3 [14, 67].

The pattern of CYP630B-CPR2 gene expression in a closely related blue-stain fungus, *Ophiostoma piceae*, very much resembles the expression profiles in *Gs*, following the same treatment with triglycerides and oleic acid [20]. This comparison across species, which inhabit a similar micro-environment and occupy similar ecological niches, indicates that this CYP system could be an important alternative in fatty acid degradation. The ω-hydroxylation pathway is possibly a concurrent free fatty acid-removal mechanism when the major ß-oxidation pathways of fatty acids are ineffective or overwhelmed by high levels of potential substrates. Such would likely be the case when the fungus encounters saturating levels of toxic plant defense monoterpenes, triglycerides and free fatty acids, all of which it must process and channel into mitochondrial and peroxisomal ß-oxidation. We offer a pathway model for the role of *Gs*CYP630B18 and fatty acid ω-hydroxylation in *Gs* during host colonization (Fig. 5). The high specificity of *Gs*CYP630 for oleic acid hydroxylation and the considerable induction of the *Gs*CYP630B18-CPR2 gene cluster following growth on triglycerides and oleic acid (Table 3), as well as its genomic organization suggest that this CYP system is an important detoxification mechanism for the removal of free oleic acid in this conifer-attacking fungus.

**Table 3:** Changes in transcript abundance of genes involved in the *Gs*CYP630B18 putative fatty acid oxidation pathway following growth on monoterpenes, triglycerides or oleic acid as the sole carbon sources.

*Gs* Gene ID	Enzyme	TG+YNB	OA+YNB	MT+YNB
CMQ_6790	LpL	322.87	1101.48	-2.11*
CMQ_2877	GK	28.53	17.50	-1.13*
CMQ_4361	ACSL	8.99	22.73	2.08
CMQ_188	FATP	2.14	2.12	-1.10*
CMQ_2411	FATP	2.01	3.64	1.23*
CMQ_4724	ACL	4.10	2.27	4.63
CMQ_1125	ACL	2.82	2.21	3.42
CMQ_6959	ACL	8.76	14.11	1107.00
CMQ_1282	CT	6.03	11.87	10.70
CMQ_2904	FOX2	9.09	31.50	1.18*
CMQ_5365	CYP630B18	326.11	14.21	3.88
CMQ_5370	CPR2	417.97	28.88	3.28

RNA-Seq data for selected genes from mycelia grown for 10 days on YNB minimal medium with a mixture of monoterpenes (YNB+MT) (monoterpenes: (+)-limonene, (+)-3-carene, racemic α-pinene and (−)-β-pinene at a ratio of 5:3:1:1), for 5 days with triglycerides (YNB+TG), or oleic acid (YNB+OA) as the sole carbon source, relative to controls grown on mannose [14, 67].

*P-values are not significant.

LpL—lipoprotein lipase, GK—glycerol kinase, ACSL—long-chain fatty acyl-CoA synthetase, FATPs—fatty acid transporter proteins, ACLs—acyl-CoA coenzymes, CT—carnitine acetyl transferase, FOX2—multifunctional β-oxidation enzyme, CYP630B18—cytochrome P450, CPR2—cytochrome P450 reductase 2.

### Modeling supports substrate specificity of GsCYP630 and its preferential use of GsCPR2

To comprehensively interpret the functioning of the GsCYP630B18-GsCPR2 redox system, we constructed a real-scale 3D-visualization of (a) the complex, anchored in the endoplasmic reticulum and (b) its interaction with oleic acid. Our main aim was to check steric requirements together with the allowed orientation to meet structural restrictions of interacting molecules. Therefore, we conducted the sequence of tasks to recognize the probable interacting surface of the GsCPR2 FMN-domain with its counterpart on the surface of GsCYP630B18. In our reasoning we included the fact that only two different CPRs exist in *Gs*. Together, they provide reducing equivalents to over fifty GsCYPs, including GsCYP630B18. We also took into account our finding that the fastest hydroxylation of highly hydrophobic oleic acid was achieved in the presence of membranes with app. 30% cholesterol and easy access to GsCPR2.

**Fig 6:**
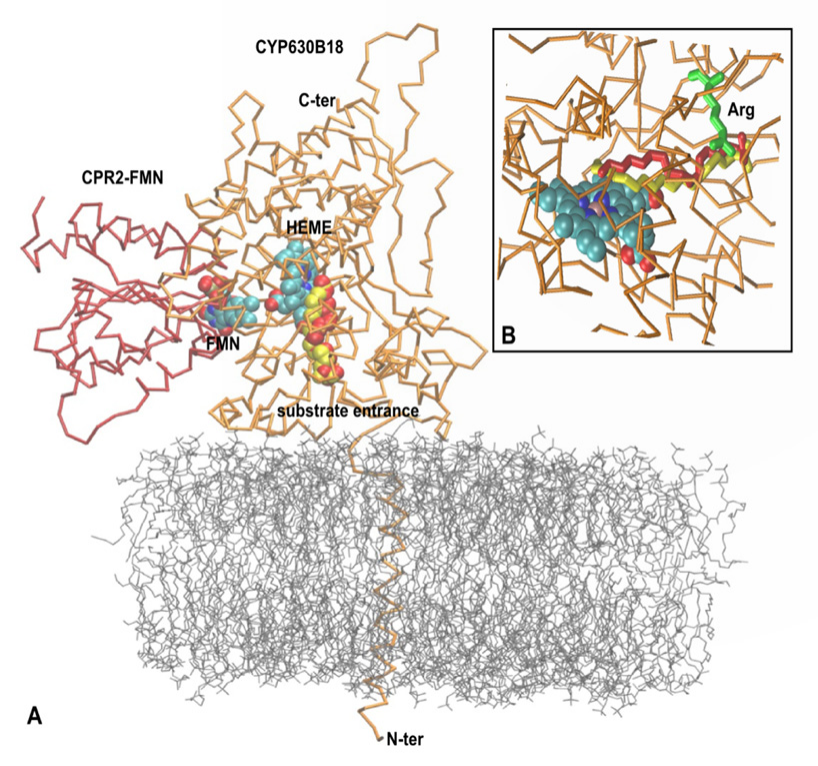
The model of RS2 reconstitution system of the FMN domain (red) of *Gs*CPR2 and *Gs*CYP630B18 (orange), its substrate oleic acid (red) and the non-substrate stearic acid (yellow). (A) The FMN-binding domain of *Gs*CPR2 in open conformation interacts with the *Gs*CYP630B18 that brings the FMN cofactor in close proximity to the CYP heme cofactor and thus facilitates electron transfer. The *Gs*CYP630B18 transmembrane region is inserted into the lipid bilayer. (B) Docking of oleic acid (red) versus the stearic acid (yellow) in the active site of *Gs*CYP630B18. Arginine 224 (green) holds both compounds in the active site channel.

Using our model, we can explain the specificity of *Gs*CYP630B18 for oleic acid among the many tested structurally different candidates (Fig. 6). We may also explain why the reaction specificity of *Gs*CYP630B18 is limited to a single oxidation reaction, and does not catalyze further oxidations of the initial reaction product, as has been reported for some ω-hydroxylating fungal CYPs [39]. For example, CYP52A13 and CYP52A17 of *Candida tropicalis* oxidize the ω-hydroxy products to fatty aldehyde and diacids without the intervention of other enzymes [68]. In contrast, with *Gs*CYP630B18 we observed no accumulation of fatty aldehyde or diacid products, suggesting that the structure of *Gs*CYP630B18 is optimized to accommodate the naturally abundant mono-desaturated C_18_ oleic acid.

**Fig 7:**
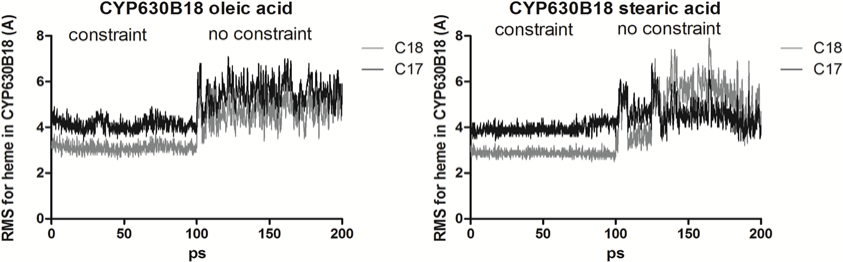
Time course of the root mean square (RMS) changes of the heme moiety in *Gs*CYP630B18 with oleic acid (left) and stearic acid (right) as a substrate. The protein was constrained for the first 100 ps.

We suggest that nonpolar oleic acid enters the CYP active site through the lipid bilayer, as it has already been shown for hydrophobic substrates that are initially recognized by the hydrophobic patch of amino acids adjacent to the substrate access channel on the membrane-associated surface of the protein [69, 70]. According to this scenario, oleic acid enters the buried active site with its ω-methyl group, while the carboxyl group interacts with Arg256 situated at the entrance. In the absence of the substrate, the positive charge of Arg256 is compensated by the electrostatic interaction with Asp137. This positioning of the substrate would allow for (a) a better steric accommodation of the ω-methyl group of mono-desaturated oleic acid over the iron atom of the heme prosthetic group in comparison to, e.g. an unsaturated stearic acid or the shorter palmitoic acid, and (b) a favorable affinity between Arg256 and the oleic carboxyl group, which can be readily released upon substitution by the carboxyl group of Asp137. Our dynamic simulations also show that the distance between the iron and the C18 atom in oleic acid remains shorter (average 4.7 Å) than the distance between the iron and C17 (average 5.6 Å). In contrast, the distances between the atoms C18 and C17 and iron are swapped after a similar dynamic simulation with stearic acid (average 5.5 Å and 4.5 Å, respectively) (Fig. 7).

**Fig 8:**
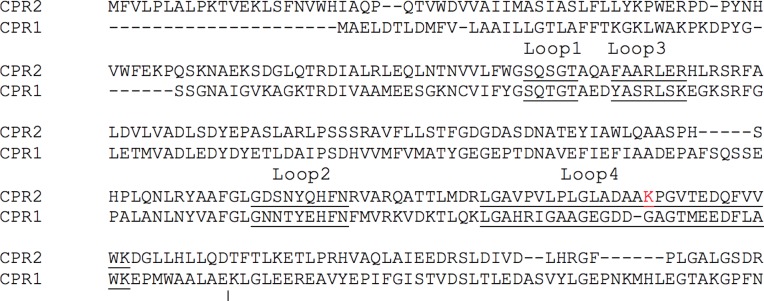
Amino acid alignment of 4 FMN-binding domains of *Gs*CPR2 and *Gs*CPR1, which interact with *Gs*CYP630B18. Loops are underlined and in bold. Lys 322 in loop 4 is marked in red.

Modeling of the protein-protein interaction revealed four loops (loop 1: S_[96]_QSGT, loop 2: T_[148]_FGDGDASDNA, loop 3: G_[186]_DSNYQHFN, loop 4: A_[221]_KPGVT) in the FMN-binding domain of *Gs*CPR2, which interacts with *Gs*CYP630B18. Very similar positioning is found in the solved crystal structure of an analogous complex between heme and FMN-binding domains of flavocytochrome P450 BM3 (PDB code: 1BVJY)—a bacterial P450 system in which a fatty acid hydroxylase CYP is fused to a eukaryotic-like CPR in a single polypeptide [71]. Loops 1–3 are comparable in both GsCPRs. Loop 1 is not comprised of any charged residues, while loops 2 and 3 can form electrostatic interactions with CYP630B18. However, considerable differences are found in loop 4. The tip of GsCPR2 loop 4 (residues 221–226) contains a charged Lys222, while the corresponding loop 4 in GsCPR1 (residues 200–224, G_[200]_GAGT) lacks positively-charged residues and is one residue shorter than its counterpart in GsCPR2 (Fig. 8).

Consequently, prior to the interaction with GsCPR2, Asp462 of GsCYP630B18 is possibly neutralized by it own Arg465. When in complex, however, Arg465 could be substituted by Lys222 of GsCPR2. In this way, much less binding energy is engaged from the electrostatic interaction, thus allowing for easier dissociation of the *Gs*CPR2 FMN domain from *Gs*CYP630B18.

### The possible function of fungal CPR paralogs

Our study further sheds light on putative roles of duplicated paralogous genes coding the typical eukaryotic microsomal cytochrome P450 partners—cytochrome P450 reductases. It has been previously shown that, unlike metazoans, plants and fungi may have more than one CPR gene in their genomes [19]. Furthermore, these duplications were independent events in the evolutionary history of plants and fungi, as well as within fungi—in zygomycetes, basidiomycetes and ascomycetes. As these two groups of eukaryotes possess the largest and most diverse CYPomes (all CYP genes per genome), it would seem convenient to have evolved more than one possible redox partner.

However, the biological roles of CPR paralogs in an organism remain unclear. The present work and earlier studies, report differences in gene expression levels of CPR under varying growth and treatment conditions, different catalytic properties and the fact that the choice of the native CPR redox partner affects CYP product specificity in *in vitro* experiments [16]. It was hypothesized that the CPR1 paralog was responsible for providing reducing equivalents to CYPs in endogenous primary metabolism, while the CPR2 possibly functions in specialized metabolism. Genomic localization and occurrence of the CPR2 paralog together with CYP630 in numerous Pezizomycotina genomes, their apparent joint gene expression profiles reported in Ophistomatales fungi grown on fatty acid substrates [14, 20], as well as functionality in oleic acid ω-hydroxylation *via* CYP630 indicate a narrower niche specialization of CPR2. However, this evidence does not rule out possible involvement of CPR2 as an alternative redox partner to other fungal CYPs found in a particular genome.

### Evolutionary conservation of the CYP630-CPR2 gene cluster

Despite the fact that genomic sequencing efforts are unevenly distributed across fungal taxa, our investigation suggests that the cluster was present in the common ancestor of the fungal classes where the cluster was retained—but not the common ancestor of Pezizomycotina. In the genome of the last common ancestor, the topological orientation of the reading frames of both genes was possibly opposite, or divergent, relative to each other on the chromosome. Multiple losses of the cluster appear to have occurred during the evolution of higher Pezizomycotina, which suggests that cluster retention might be selected against.

Clustered genes with opposite orientation are more tightly regulated and tend to be co-expressed which may be due to the close proximity of their promoter regions [72, 46]. In cases of biosynthetic gene clusters, such physical linkage in pairs of fungal genes may be an adaptation to effectively metabolize harmful pathway intermediates [47]. Although the *Gs*CYP630B18-*Gs*CPR2 cluster is not a biosynthetic cluster, its genomic organization suggests that it may contribute to efficient elimination of toxic free oleic acid. The presence of the cluster in other fungi is not correlated with taxonomy, lifestyle (saprophyte *vs*. pathogen) or ecological niche (specialists *vs*. generalists). It is possible, that cluster conservation could be required in species that need to process free C18 fatty acids, regardless of their source.

## Conclusions

In this study, we have functionally characterized a fungal CYP630 as a highly specific oleic acid ω-hydroxylase. To better understand the metabolic role of this cytochrome P450, we have considered and integrated information on its genomic co-localization with its probable redox partner, previously published relevant transcriptomic results and the chemical microenvironment that this fungus encounters during host pine tree colonization. In summary, these data suggest that this CYP system is an important mechanism for the elimination of toxic free fatty acids, and helps to counter the metabolic stress in the fungus caused by conifer chemical defenses. Further studies on transcriptional regulation, substrate specialization and the function of this evolutionarily conserved system in other filamentous ascomycetes are needed to better understand the role of the CYP630-CPR2 system in fungal pathogenesis and host-adapted metabolism.

## Supporting Information

S1 Table Strains, plasmids and primers used in this study. (PDF)

S2 Table List of amino acids added to SD-URA yeast medium. (PDF)

S3 Table The LC gradient used in enzyme reaction extract analyses. (PDF)

S4 Table Predicted values [m/z] for the fragmentation pattern of 18-hydroxyoleic acid. (PDF)

S1 Fig Extracted ion chromatograms of RS1 and RS2 products and mass spectra (MS) for stearic acid as substrate. (A) Extracted ion chromatograms run in negative-ion ESI from LC/MS analyses for GsCYP630B18 metabolic activity in RS1 and RS2 with stearic acid as substrate, and (B) MS for the 17.5 min product peak. The range of m/z was chosen to extract the masses of potential hydroxylated products of stearic acid. Empty *E*. *coli* membrane fractions were used as a control. According to similar MS patterns from control and RS experiments, the 17.5 min product peak does not represent hydroxy-acid products or further downstream intermediates as metabolites of the enzyme reactions.
(PDF)

S2 Fig Extracted ion chromatograms of RS1 and RS2 products. Extracted ion chromatograms run in negative-ion ESI from LC/MS analyses for *Gs*CYP630B18 metabolic activity in RS1 and RS2 with (A) arachidonic acid, (B) capric acid, (C) lauric acid, (D) linoleic acid, (E) myristic acid, (F) palmitic acid and (G) palmitoleic acid as substrates. Empty *E*. *coli* membrane fractions were used as control.
(PDF)

S3 Fig Fragmentation pattern of 18-hydroxyoleic acid. The compound was analyzed with an Agilent 1100 series LCMSD ion trap XCT Plus system. The fragmentation was generated by using a collision energy of 20 volts and negative ionization mode.
(PDF)
