# Supplementary material for: Oleic Acid Metabolism via a Conserved Cytochrome P450 System-Mediated ω-Hydroxylation in the Bark Beetle-Associated Fungus Grosmannia clavigera
Source: PLoS One. 2015 Mar 20;10(3):e0120119. doi: 10.1371/journal.pone.0120119 (PMC4368105; doi:10.1371/journal.pone.0120119)
Supplement: S1 Table — (PDF) [file pone.0120119.s001.pdf]

S1 Table. Strains, plasmids and primers used in this study.

	Genotype/description	Source
**Strains**		
BY4741 (*S.cerevisiae*)	*MATa his3Δ0 leu2Δ0 met15Δ0 ura3Δ0*	Open Biosystems
DH5α (*E. coli*)	*F- gyrA96 (Nalr) recA1 endA1 thi-1 hsdR17 (rk-mk-) gluV44 deoR* Φ80d Δ(lacZ)M15	[1]
C43(DE3) (*E. coli*)	*F- ompT hsdSB (rB- mB-) gal dcm* (DE3)	[2]
**Plasmids**		
pET17b-P450	Vector with *Gs* P450 gene (cDNA)	This study
pYeDP60U-CPR1	Vector with *Gs* CPR1gene (cDNA)	This study
pYeDP60U-CPR2	Vector with *Gs* CPR1*g*ene (cDNA)	This study
**Primers**	5′ → 3′ orientation	
CYP630B18-fw_*Nde*I	GACATCCATAT*G*TATGCCCTTCTGTGCCAGC	
CYP630B18-rev_*Eco*RI	GGAGGTGGAGTTGAAGGAAGAGCTCTCATCAC TGAGAATTCGATGTC	
CPR1-pYeD_*Bam*HI	CTAAATTACCGGATCATGGCGAACTGG ACACGCTGGAC	
CPR1-pYeD_ *Eco*RI	CCAATACCAGGAGGACGTTTGGTGAAATTCGC GGGGGATC	
CPR2-pYeD_*Bam*HI	CTAAATTACCGGATCATGTTTGTCC TGCCTTTGGCTCTGC	
CPR2-pYeD_ *Eco*RI	GCTATATCGAGGATGTGTGGGGTTGAAATTCG CGGGGGATC	
**PCR conditions**		
CYP6330B18	95°C/3′ 15x(95°C/30′′ (55°C/65°C)/45′′ 72°C/60′′) 25x(95°C/30′′ 55°C/45′′ 72°C/60′′) 72°C/5′	
CPR1	95°C/3′ 35x(95°C/30′′ 55°C/30′′ 72°C/45′′) 72°C/5′	
CPR2	95°C/3′ 15x(95°C/30′′ (55°C/65°C)/45′′ 72°C/60′′) 25x(95°C/30′′ 55°C/45′′ 72°C/60′′) 72°C/5′	
	95°C/3′ 14x(95°C/10′′ (62°C/72°C)/45′′ 72°C/60′′) 29x(95°C/10′′ 62°C/55′′ 72°C/60′′) 72°C/5′	

Restriction enzyme cut sites are underlined.
