# Supplementary material for: Oleic Acid Metabolism via a Conserved Cytochrome P450 System-Mediated ω-Hydroxylation in the Bark Beetle-Associated Fungus Grosmannia clavigera
Source: PLoS One. 2015 Mar 20;10(3):e0120119. doi: 10.1371/journal.pone.0120119 (PMC4368105; doi:10.1371/journal.pone.0120119)
Supplement: S2 Table — (PDF) [file pone.0120119.s002.pdf]

S2 Table. List of amino acids added to SD-URA yeast medium.

Amino acid*	Concentration
Adenine hemisulfate	1 g/l
Argenine	5 g/l
Histidine	2 g/l
Leucine	10 g/l
Lysine	5 g/l
Methionine	2 g/l
Phenylalanine	5 g/l
Threonine	30 g/l
Tryptophan	5 g/l

*All chemicals purchased from Sigma-Aldrich (Germany).
