# Supplementary material for: Oleic Acid Metabolism via a Conserved Cytochrome P450 System-Mediated ω-Hydroxylation in the Bark Beetle-Associated Fungus Grosmannia clavigera
Source: PLoS One. 2015 Mar 20;10(3):e0120119. doi: 10.1371/journal.pone.0120119 (PMC4368105; doi:10.1371/journal.pone.0120119)
Supplement: S3 Table — (PDF) [file pone.0120119.s003.pdf]

S3 Table. The LC gradient used in enzyme reaction extract analyses.

Time (min)	Water (%)	Acetonitrile (%)
0	40	60
15	20	80
17.5	15	85
18	5	95
26	5	95
27	0	100
33	0	100
34	40	60
40	40	60
