# Supplementary material for: Oleic Acid Metabolism via a Conserved Cytochrome P450 System-Mediated ω-Hydroxylation in the Bark Beetle-Associated Fungus Grosmannia clavigera
Source: PLoS One. 2015 Mar 20;10(3):e0120119. doi: 10.1371/journal.pone.0120119 (PMC4368105; doi:10.1371/journal.pone.0120119)
Supplement: S4 Table — (PDF) [file pone.0120119.s004.pdf]

S4 Table. Predicted values [m/z] for the fragmentation pattern of 18-hydroxyoleic acid.

[298] C18H34O3 (1)	[237] C15H25O2 (15)	[208] C15H28 (10)	[180] C12H20O (5)	[152] C10H16O (5)
[297] C18H33O3 (19)	[236] C17H32 (5)	[208] C14H24O (5)	[179] C13H23 (7)	[151] C11H19 (9)
[296] C18H32O3 (182)	[236] C16H28O (5)	[207] C15H27 (5)	[179] C12H19O (3)	[151] C10H15O (3)
[281] C18H33O2 (2)	[235] C17H31 (1)	[207] C14H23O (3)	[178] C13H22 (41)	[150] C11H18 (55)
[280] C18H32O2 (29)	[235] C16H27O (3)	[206] C15H26 (31)	[178] C12H18O (29)	[150] C10H14O (29)
[279] C18H31O2 (69)	[234] C17H30 (8)	[206] C14H22O (28)	[169] C11H21O (23)	[143] C9H19O (1)
[278] C18H30O2(424)	[234] C16H26O (29)	[197] C13H25O (14)	[169] C10H17O2 (10)	[143] C8H15O2 (8)
[267] C17H31O2 (17)	[225] C15H29O (16)	[197] C12H21O2 (12)	[168] C11H20O (19)	[142] C9H18O (5)
[266] C17H30O2 (14)	[225] C14H25O2 (14)	[196] C13H24O (15)	[168] C10H16O2 (14)	[142] C8H14O2 (13)
[265] C17H29O2 (17)	[224] C15H28O (15)	[196] C12H20O2 (15)	[167] C12H23 (6)	[141] C9H17O (1)
[263] C18H31O (4)	[224] C14H24O2 (15)	[195] C14H27 (4)	[167] C11H19O (24)	[141] C8H13O2 (8)
[262] C18H30O (35)	[223] C16H31 (1)	[195] C13H23O (15)	[167] C10H15O2 (9)	[140] C9H16O (5)
[253] C17H33O (18)	[223] C15H27O (17)	[195] C12H19O2 (12)	[166] C12H22 (30)	[139] C10H19 (8)
[253] C16H29O2 (16)	[223] C14H23O2 (14)	[194] C14H26 (20)	[166] C11H18O (10)	[138] C10H18 (40)
[252] C17H32O (5)	[222] C16H30 (5)	[194] C13H22O (5)	[165] C12H21 (8)	[137] C10H17 (10)
[252] C16H28O2 (15)	[222] C15H26O (5)	[193] C14H25 (6)	[165] C11H17O (4)	[136] C10H16 (58)
[251] C17H31O (19)	[221] C16H29 (4)	[193] C13H21O (3)	[164] C12H20 (47)	[129] C8H17O (9)
[251] C16H27O2 (16)	[221] C15H25O (3)	[192] C14H24 (37)	[164] C11H16O (42)	[129] C7H13O2 (7)
[250] C17H30O (5)	[220] C16H28 (26)	[192] C13H20O (29)	[157] C9H17O2 (1)	[128] C8H16O (13)
[249] C17H29O (3)	[220] C15H24O (30)	[183] C12H23O (13)	[156] C9H16O2 (5)	[128] C7H12O2 (12)
[248] C17H28O (29)	[211] C14H27O (15)	[183] C11H19O2 (21)	[155] C10H19O (11)	[127] C8H15O (10)
[239] C16H31O (1)	[211] C13H23O2 (13)	[182] C12H22O (15)	[155] C9H15O2 (1)	[127] C7H11O2 (7)
[239] C15H27O2 (15)	[210] C14H26O (15)	[182] C11H18O2 (19)	[154] C10H18O (13)	[126] C8H14O (7)
[238] C16H30O (15)	[210] C13H22O2 (15)	[181] C13H25 (5)	[154] C9H14O2 (5)	[125] C9H17 (8)
[238] C15H26O2 (15)	[209] C15H29 (2)	[181] C12H21O (14)	[153] C11H21 (8)	[125] C8H13O (3)
[237] C17H33 (1)	[209] C14H25O (16)	[181] C11H17O2 (20)	[153] C10H17O (11)	[124] C9H16 (49)
[237] C16H29O (18)	[209] C13H21O2 (13	[180] C13H24 (25)	[152] C11H20 (40)	[124] C8H12O (26)
[123] C9H15 (8)	[100] C5H8O2 (9)	[84] C5H8O (2)	[67] C5H7 (4)	[43] C2H3O (3)
[122] C9H14 (9)	[99] C7H15 (1)	[83] C6H11 (5)	[59] C3H7O (4)	[42] C3H6 (2)
[115] C7H15O (8)	[99] C6H11O (8)	[83] C5H7O (6)	[59] C2H3O2 (4)	[42] C2H2O (2)
[115] C6H11O2 (6)	[99] C5H7O2 (5)	[82] C6H10 (8)	[58] C3H6O (6)	[41] C3H5 (3)
[114] C7H14O (12)	[98] C7H14 (1)	[82] C5H6O (11)	[58] C2H2O2 (3)	[41] C2HO (1)
[114] C6H10O2 (10)	[98] C6H10O (2)	[81] C6H9 (5)	[57] C4H9 (1)	[40] C3H4 (5)
[113] C8H17 (1)	[97] C7H13 (7)	[73] C4H9O (5)	[57] C3H5O (6)	[39] C3H3 (2)
[113] C7H13O (9)	[97] C6H9O (7)	[73] C3H5O2 (3)	[56] C4H8 (3)	[31] CH3O (2)
[113] C6H9O2 (6)	[96] C7H12 (14)	[72] C4H8O (8)	[56] C3H4O (3)	[30] CH2O (1)
[112] C8H16 (1)	[96] C6H8O (16)	[72] C3H4O2 (4)	[55] C4H7 (4)	[29] CHO (1)
[112] C7H12O (2)	[95] C7H11 (6)	[71] C5H11 (1)	[55] C3H3O (2)	[28] C2H4 (1)
[111] C8H15 (8)	[87] C5H11O (6)	[71] C4H7O (7)	[54] C4H6 (12)	[27] C2H3 (1)
[111] C7H11O (5)	[87] C4H7O2 (4)	[71] C3H3O2 (3)	[54] C3H2O (4)	[26] C2H2 (1)
[110] C8H14 (17)	[86] C5H10O (10)	[70] C5H10 (1)	[53] C4H5 (3)	[25] C2H (1)
[110] C7H10O (16)	[86] C4H6O2 (5)	[70] C4H6O (5)	[45] C2H5O (3)	[17] HO (1)
[109] C8H13 (7)	[85] C6H13 (1)	[69] C5H9 (5)	[45] CHO2 (2)	[15] CH3 (1)
[101] C6H13O (7)	[85] C5H9O (7)	[69] C4H5O (5)	[44] C2H4O (3)	
[101] C5H9O2 (5)	[85] C4H5O2 (4)	[68] C5H8 (9)	[44] CO2 (2)	
[100] C6H12O (11)	[84] C6H12 (1)	[68] C4H4O (7)	[43] C3H7 (1)	

Numbers of possible fragment ion structures with the specific molecular weights [m/z] are in brackets.
