# Supplementary material for: Oleic Acid Metabolism via a Conserved Cytochrome P450 System-Mediated ω-Hydroxylation in the Bark Beetle-Associated Fungus Grosmannia clavigera
Source: PLoS One. 2015 Mar 20;10(3):e0120119. doi: 10.1371/journal.pone.0120119 (PMC4368105; doi:10.1371/journal.pone.0120119)

S1 Fig. Extracted ion chromatograms of RS1 and RS2 products and mass spectra (MS) for stearic acid as substrate. (A) Extracted ion chromatograms run in negative-ion ESI from LC/MS analyses for GsCYP630B18 metabolic activity in RS1 and RS2 with stearic acid as substrate, and (B) MS for the 17.5 min product peak. The range of m/z was chosen to extract the masses of potential hydroxylated products of stearic acid. Empty *E. coli* membrane fractions were used as a control. According to similar MS patterns from control and RS experiments, the 17.5 min product peak does not represent hydroxy-acid products or further downstream intermediates as metabolites of the enzyme reactions.

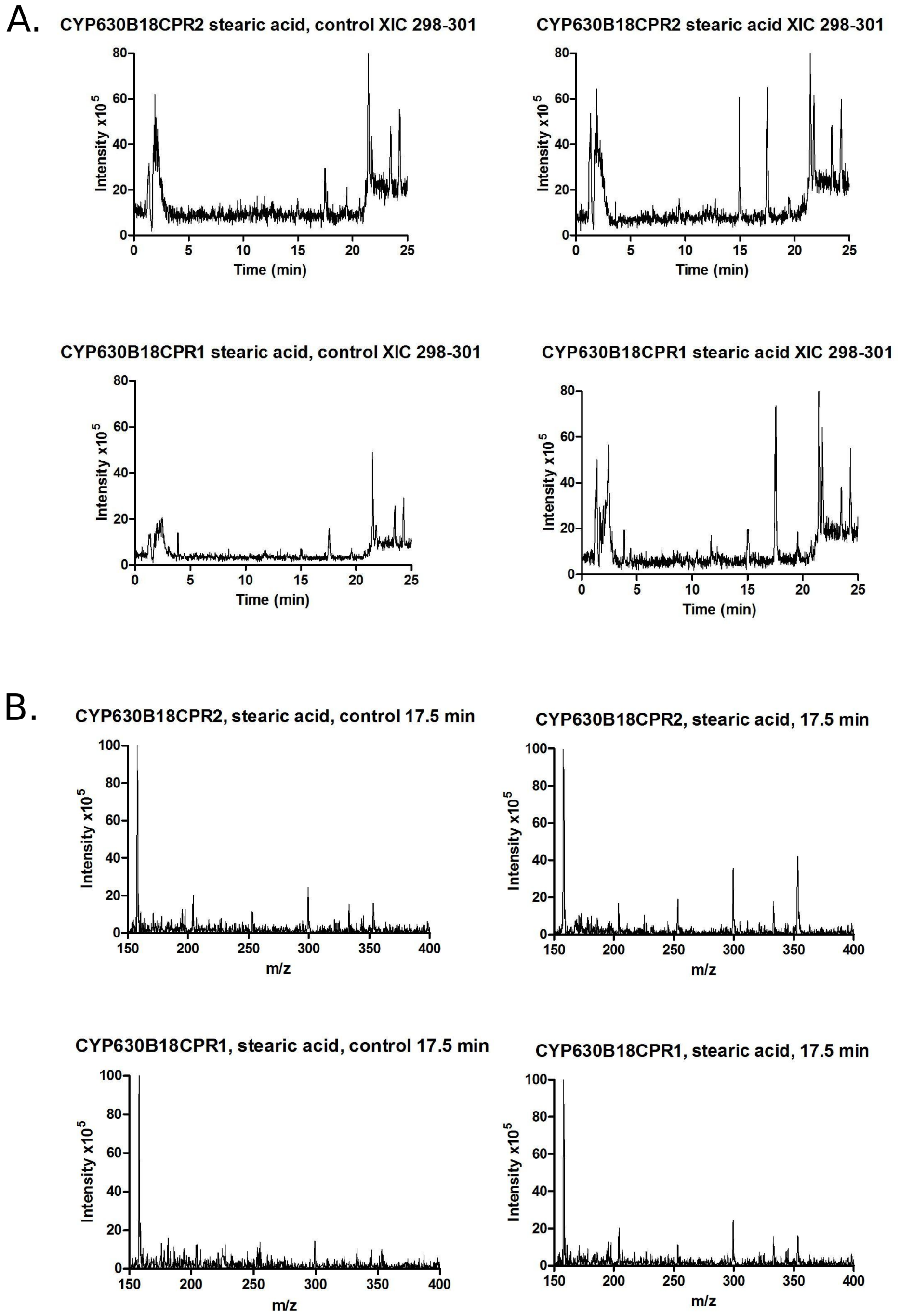

Supplement: S1 Fig — (A) Extracted ion chromatograms run in negative-ion ESI from LC/MS analyses for GsCYP630B18 metabolic activity in RS1 and RS2 with stearic acid as substrate, and (B) MS for the 17.5 min product peak. The range of m/z was chosen to extract the masses of potential hydroxylated products of stearic acid. Empty E. coli membrane fractions were used as a control. According to similar MS patterns from control and RS experiments, the 17.5 min product peak does not represent hydroxy-acid products or further downstream intermediates as metabolites of the enzyme reactions. (PDF) [file pone.0120119.s005.pdf]
