# Supplementary material for: Oleic Acid Metabolism via a Conserved Cytochrome P450 System-Mediated ω-Hydroxylation in the Bark Beetle-Associated Fungus Grosmannia clavigera
Source: PLoS One. 2015 Mar 20;10(3):e0120119. doi: 10.1371/journal.pone.0120119 (PMC4368105; doi:10.1371/journal.pone.0120119)
Supplement: S2 Fig — Extracted ion chromatograms run in negative-ion ESI from LC/MS analyses for GsCYP630B18 metabolic activity in RS1 and RS2 with (A) arachidonic acid, (B) capric acid, (C) lauric acid, (D) linoleic acid, (E) myristic acid, (F) palmitic acid and (G) palmitoleic acid as substrates. Empty E. coli membrane fractions were used as control. (PDF) [file pone.0120119.s006.pdf]

S2 Fig. Extracted ion chromatograms of RS1 and RS2 products. Extracted ion chromatograms run in negative-ion ESI from LC/MS analyses for *Gs*CYP630B18 metabolic activity in RS1 and RS2 with (A) arachidonic acid, (B) capric acid, (C) lauric acid, (D) linoleic acid, (E) myristic acid, (F) palmitic acid and (G) palmitoleic acid as substrates. Empty *E. coli* membrane fractions were used as control.

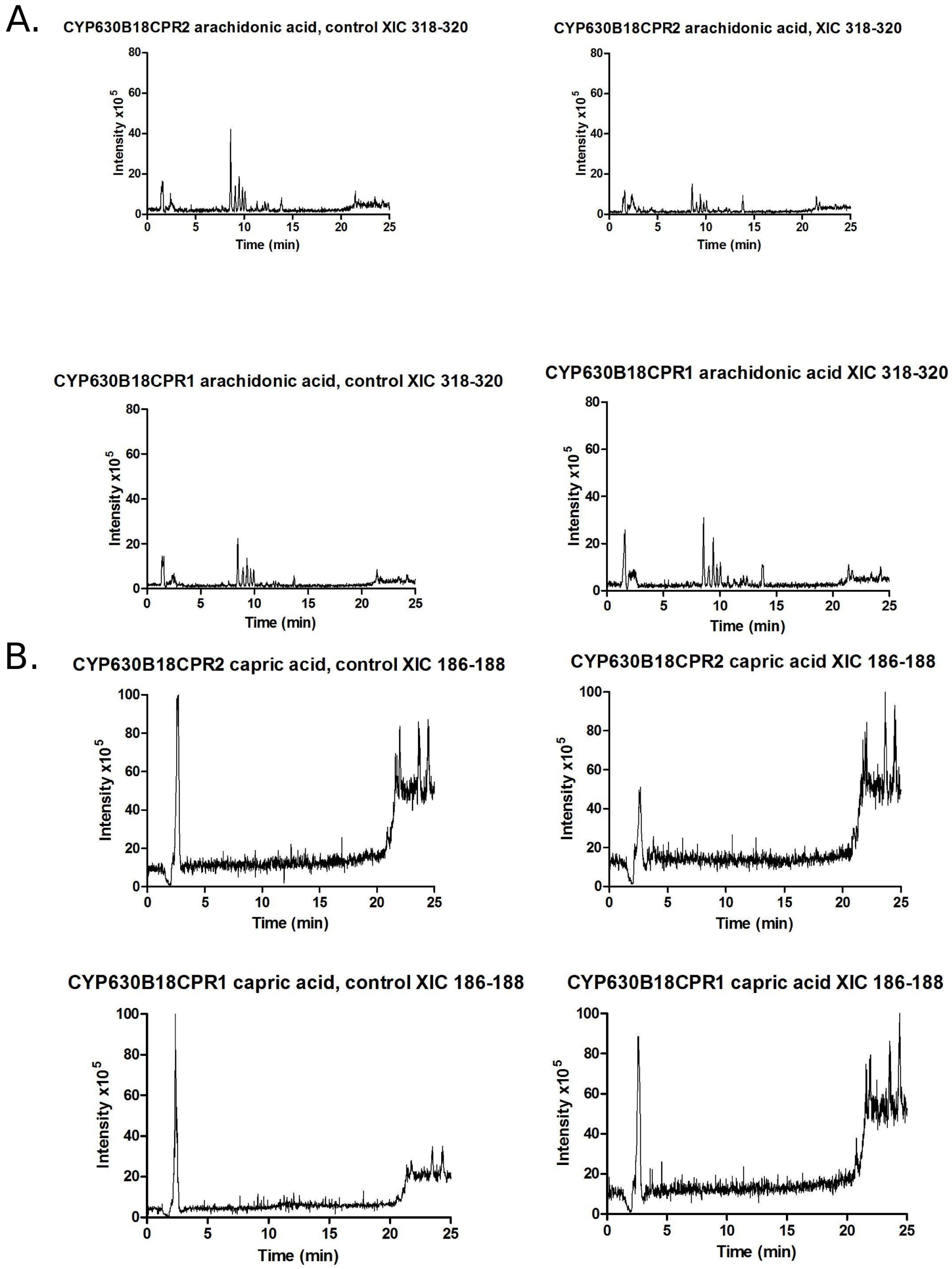

C.

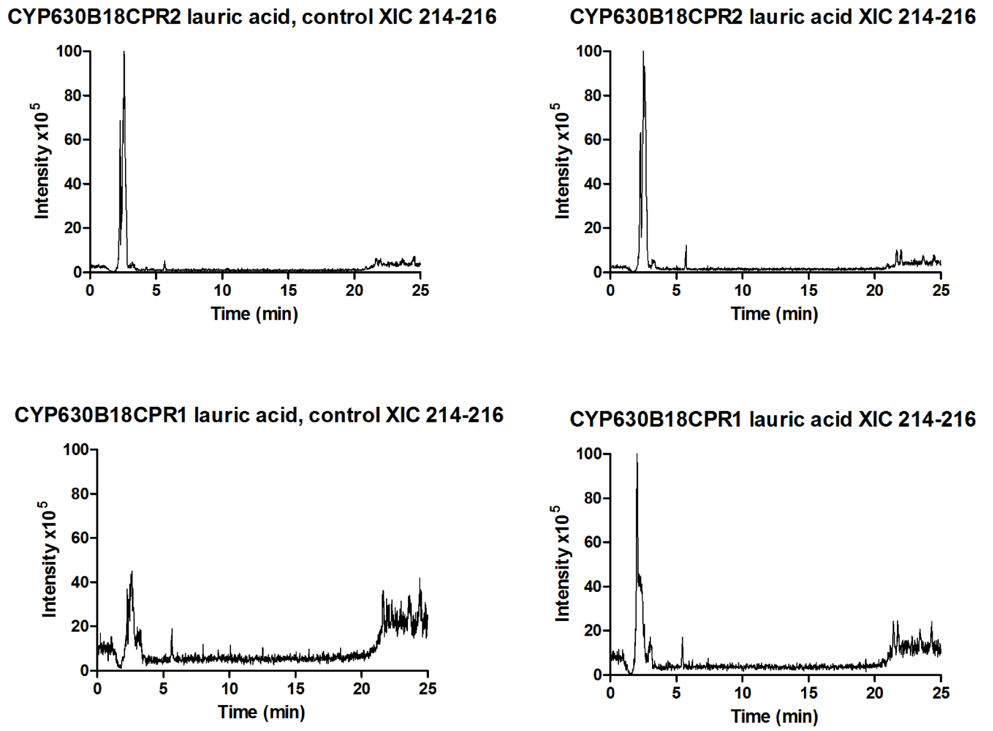
CYP630B18CPR2 lauric acid, control XIC 214-216
CYP630B18CPR2 lauric acid XIC 214-216
CYP630B18CPR1 lauric acid, control XIC 214-216
CYP630B18CPR1 lauric acid XIC 214-216
Intensity x10^5
Time (min)
0
5
10
15
20
25
20
40
60
80
100

D.

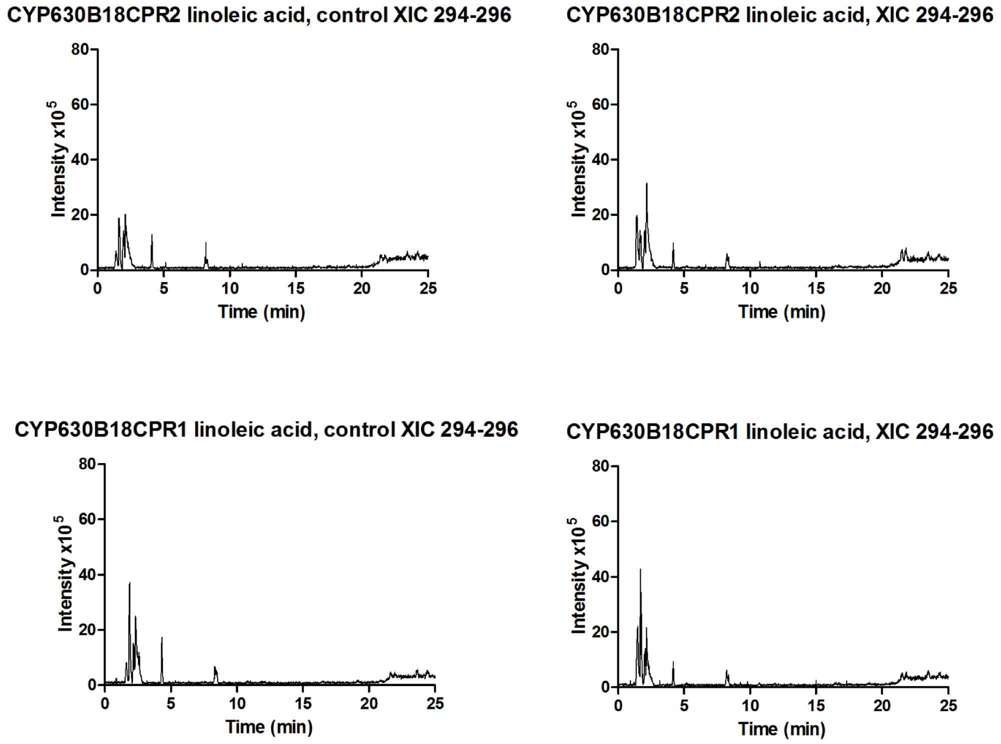
CYP630B18CPR2 linoleic acid, control XIC 294-296
CYP630B18CPR2 linoleic acid, XIC 294-296
CYP630B18CPR1 linoleic acid, control XIC 294-296
CYP630B18CPR1 linoleic acid, XIC 294-296
Intensity x10^5
Time (min)
0
5
10
15
20
25
20
40
60
80

E.

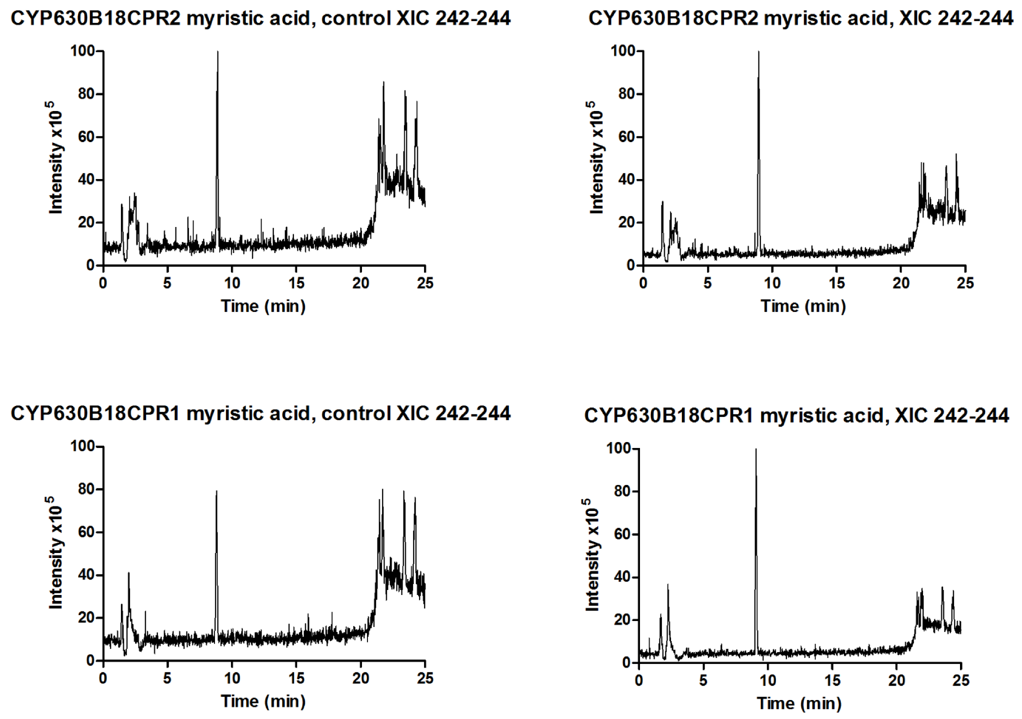
CYP630B18CPR2 myristic acid, control XIC 242-244
CYP630B18CPR2 myristic acid, XIC 242-244
CYP630B18CPR1 myristic acid, control XIC 242-244
CYP630B18CPR1 myristic acid, XIC 242-244
Intensity x10^5
Time (min)

F.

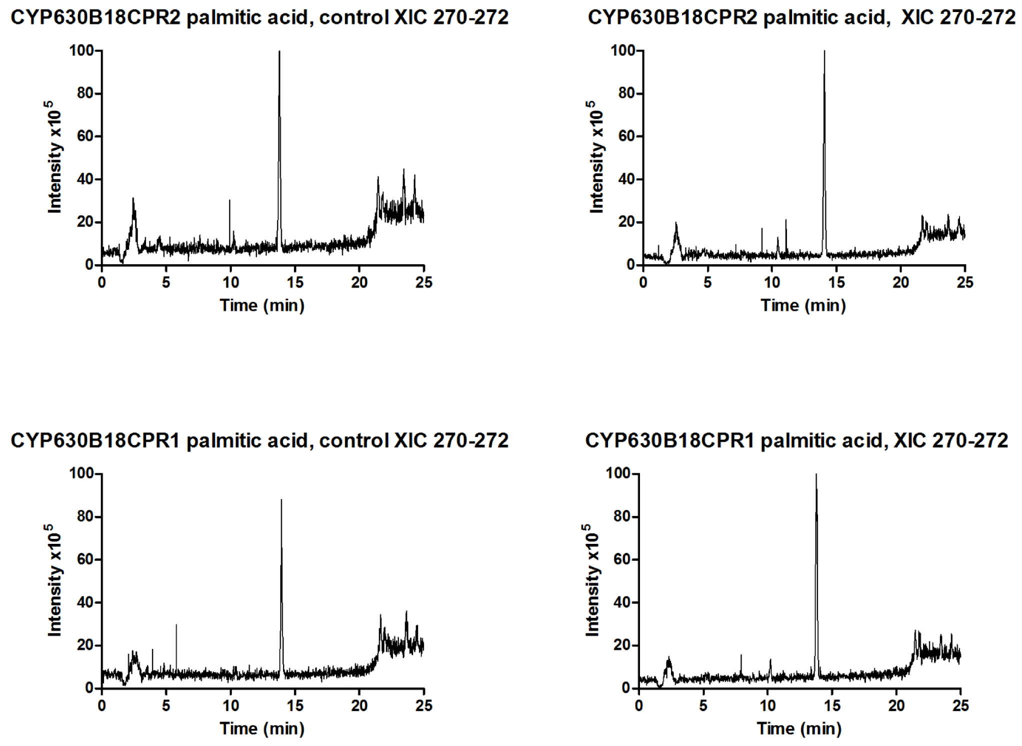
CYP630B18CPR2 palmitic acid, control XIC 270-272
CYP630B18CPR2 palmitic acid, XIC 270-272
CYP630B18CPR1 palmitic acid, control XIC 270-272
CYP630B18CPR1 palmitic acid, XIC 270-272
Intensity x10^5
Time (min)

G.

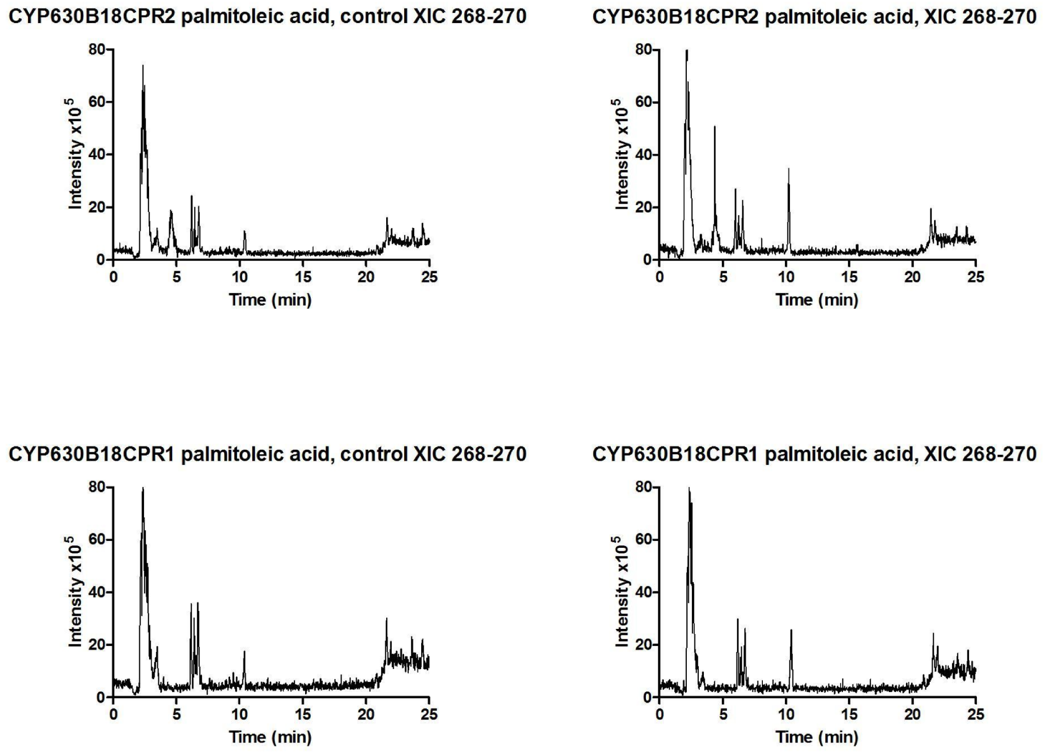
CYP630B18CPR2 palmitoleic acid, control XIC 268-270
CYP630B18CPR2 palmitoleic acid, XIC 268-270
CYP630B18CPR1 palmitoleic acid, control XIC 268-270
CYP630B18CPR1 palmitoleic acid, XIC 268-270
Intensity x10^5
Time (min)
0
20
40
60
80
0
5
10
15
20
25
