# Supplementary material for: Oleic Acid Metabolism via a Conserved Cytochrome P450 System-Mediated ω-Hydroxylation in the Bark Beetle-Associated Fungus Grosmannia clavigera
Source: PLoS One. 2015 Mar 20;10(3):e0120119. doi: 10.1371/journal.pone.0120119 (PMC4368105; doi:10.1371/journal.pone.0120119)

S3 Fig. Fragmentation pattern of 18-hydroxyoleic acid. The compound was analyzed with an Agilent 1100 series LCMSD ion trap XCT Plus system. The fragmentation was generated by using a collision energy of 20 volts and negative ionization mode.

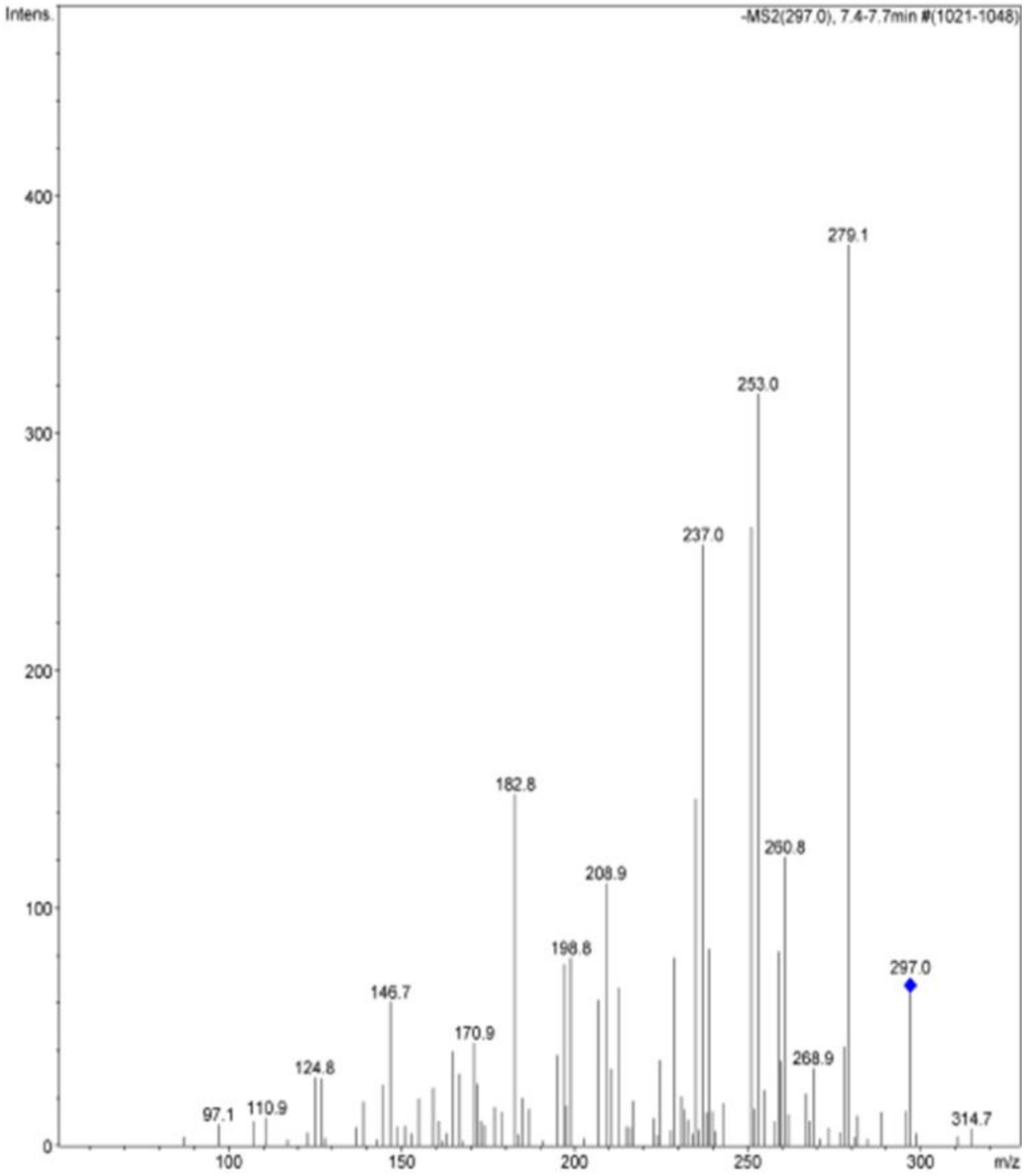

Supplement: S3 Fig — The compound was analyzed with an Agilent 1100 series LCMSD ion trap XCT Plus system. The fragmentation was generated by using a collision energy of 20 volts and negative ionization mode. (PDF) [file pone.0120119.s007.pdf]
